# Whole genome sequence characterization of *Aspergillus terreu*s ATCC 20541 and genome comparison of the fungi *A. terreu*s

**DOI:** 10.1038/s41598-022-27311-7

**Published:** 2023-01-05

**Authors:** Hung-Yi Wu, Uffe Hasbro Mortensen, Fang-Rong Chang, HsinYuan Tsai

**Affiliations:** 1 Department of Marine Biotechnology and Resources, National Sun Yat-Sen University, Kaohsiung City, Taiwan; 2 DTU Bioengineering, Technical University of Denmark, 2800 Kgs. Lyngby, Denmark; 3 Graduate Institute of Natural Products, College of Pharmacy, Kaohsiung Medical University, Kaohsiung City, Taiwan; 4 Doctoral Degree Program in Marine Biotechnology, National Sun Yat-Sen University, Kaohsiung City, Taiwan

**Keywords:** Microbial genetics, Microbiology

## Abstract

*Aspergillus terreus* is well-known for lovastatin and itaconic acid production with biomedical and commercial importance. The mechanisms of metabolite formation have been extensively studied to improve their yield through genetic engineering. However, the combined repertoire of carbohydrate-active enzymes (CAZymes), cytochrome P450s (CYP) enzymes, and secondary metabolites (SMs) in the different *A. terreus* strains has not been well studied yet, especially with respect to the presence of biosynthetic gene clusters (BGCs). Here we present a 30 Mb whole genome sequence of *A. terreus* ATCC 20541 in which we predicted 10,410 protein-coding genes. We compared the CAZymes, CYPs enzyme, and SMs across eleven *A. terreus* strains, and the results indicate that all strains have rich pectin degradation enzyme and CYP52 families. The lovastatin BGC of *lovI* was linked with *lovF* in *A. terreus* ATCC 20541, and the phenomenon was not found in the other strains. *A. terreus* ATCC 20541 lacked a non-ribosomal peptide synthetase (AnaPS) participating in acetylaszonalenin production, which was a conserved protein in the ten other strains. Our results present a comprehensive analysis of CAZymes, CYPs enzyme, and SM diversities in *A*. *terreus* strains and will facilitate further research in the function of BGCs associated with valuable SMs.

## Introduction

*Aspergillus* spp. have the ability to adapt to various environments, likely, in part, because they produce a large array of secondary metabolites (SMs)^1^. SMs can be used in survival strategies against competitors via acidification, antifungal, and antiparasitic activity in nature and for invading hosts^2,3^. Since fungi represent a vast resource for the discovery of new valuable carbohydrate-active enzymes (CAZymes) and SMs^2,4^, there is a massive interest in sequencing new species and species isolates with the aim of finding new genes and products^5^. To this end, we note that the identification of genes for SM production is less complicated in fungi as all genes relevant for the production of a given SM are typically organized in biosynthetic gene clusters (BGCs), rather than being scattered around in the genome like in plants^6,7^. Typically, fungal BGCs contain a gene coding for a synthase that supplies the scaffold, which could be a non-ribosomal peptide, a terpene, or a polyketide; and genes coding for tailoring enzymes, which decorate the scaffold to produce the mature SM^8^.

*Aspergillus terreus* is commonly found in many diverse habitats including agricultural, mangrove, soil rhizospheres in tropical and subtropical regions, organs of living creatures, and even in the human lungs and sputum^9–11^. Importantly, *A. terreus* is a harmful pathogen to crops, which has been reported in rice (*Oryza sativa* L.), wheat (*Triticum aestivum*), potato (*Solanum tuberosum* L.), maize (*Zea mays*), and soybean (*Glycine max* L.)^12,13^, and it may even infect humans to cause detrimental invasive aspergillosis (IA)^14,15^. Though *A. terreus* causes severe agricultural damage and acts as a human pathogen, it is also a well-known industrial workhorse. For example, it is a well-known producer of itaconic acid^16,17^ and lovastatin^18,19^, the former of which is heavily used in the chemical industries, and the latter of which is an important cholesterol-lowering drug. *A. terreus* also produce many carbohydrate-active enzymes (CAZymes) including cellulases, xylanases, lipases, and phytases that are employed in plant biomass degradation^20–24^.

Given the large interest in *A. terreus*, ten whole genome sequences of *A. terreus* strains are available in the National Center for Biotechnology Information Search database (NCBI). This includes the whole genome of *A. terreus* NIH2624, which was the first reference genome for *A. terreus*. The genome of *A. terreus* strains M6925, ATCC 20542, ML-44, ASM-1, TN-484, IFO 6365, w25, 45A, and T3 Kankrej were subsequently published from 2016 to 2021^25–30^. Many fungal genomes have been included in comparative studies aiming at improving the understanding of SM production, and discovering the evolutionary patterns in SM gene clusters diversity in fungal species, e.g., *Aspergillus* species (*A. fumigatus*, *A. nidulans*, and *A. flavus*), *Fusarium* species (*F. oxysporum* and *F. fujikuroi*) and *Botrytis cinerea*^31–37^. However, comprehensive comparisons of SM gene cluster polymorphisms of selected *A*. *terreus* strains have not be characterized.

In this study, we provide a thorough overview of the natural product repertoire of *A. terreus*. One future goal is to improve the yield of lovastatin and genomic differences may contribute useful insights. Towards this goal, we sequenced the genome of *A. terreus* isolate, ATCC 20541. We then compared all eleven *A. terreus* whole genomes, including genes encoding putative CAZymes, cytochrome P450s (CYPs), and BGC for SM production, which help to understand genomic diversity between *A. terreus* ATCC 20541 and other *A. terreus* strains. In particular, the variations in SM BGCs within *A. terreus* strains were analyzed. Overall, this study provides a high-quality whole genome assembly and annotation of *A. terreus* ATCC 20541 and a comprehensive analysis of CAZymes, CYPs, and SMs diversity in *A. terreus* strains.

## Results

### Assembly statistics and general features of *A. terreus* ATCC 20541 genome

We first fully genome sequenced *A. terreus* ATCC 20541 using Novaseq 6000 platforms (Illumina, USA) with 2 × 150 paired-end reads and obtained a total number of 69.7 million reads (~ 349 × coverage). The assembled genome represents 97.6% completeness (BUSCO) with a contig N50 length of 1.6 Mb and the longest contig of 3.8 Mb. The resulting genome assembly was based on 156 contigs with a 52.3% GC content, 10,410 protein-coding genes, 33 rRNA genes, and 180 tRNA genes. 1016 signal peptides and 2483 transmembrane helices were detected. Pfam domains were assigned to 8235 proteins based on InterProscan program. Subsequently, a total of 6689 proteins were assigned to the eukaryotic orthologous groups (KOG) databases (Table 1; Supplementary Fig. S1). The abundance of metabolism (III) was about 60%, which was the highest in these four categories. 2053 genes (19.7%) were annotated using KAAS and KEGG map, and KEGG annotations contained seven major pathways including BRITE hierarchies (36%), metabolism (34%), genetic information processing (13%), cellular processes (8%), environmental information processing (6%), human pathogenicity (2%), and organismal systems (2%). 18 genes were classified into the biosynthesis of “other” secondary metabolites (Supplementary Fig. S2). Gene ontology (GO) terms were divided into three major function categories: biological processes (39%), molecular functions (35%), and cellular components (26%). 8,671 genes (83%) were annotated and assigned to the three categories. 185 genes were classified into the secondary metabolite biosynthetic process (Supplementary Fig. S3; Supplementary Table S1). 1434 genes were unknown function in KOG, GO, and KEGG.

**Table 1:** Genomic features of the *A. terreus* ATCC 20541.

Features	Value
Total number of reads-Illumina (bp)	69,681,640
Genome coverage-Illumina	349 X
Contigs/scaffolds	156
Largest contigs/scaffolds (bp)	3,819,325
N50 (bp)	1,581,806
Complete BUSCOs	97.6%
Number of protein coding genes	10,410
rRNA genes	33
tRNA genes	180
Proteins with signal peptide	1016
Proteins with transmembrane helices	2483
Proteins with predicted Pfam domain	8235
Proteins assigned to KOG	6689
Predicted CAZymes proteins	479

### Phylogenetic tree and comparative genomics of *A. terreus* strains

In addition to the novel sequence of *A*. *terreus* ATCC 20541, we obtained the genomes of ten additional *A. terreus* strains accessed from the NCBI database. Previous research reported that *A. terreus* can be isolated in many different environments, including humans, soil, plant root, land, and marine creatures (Supplementary Fig. S4). Based on this, we found that the average genome size of the *A. terreus* strains was about 29.8 Mb and the average GC content was 52.5%. We observed three main clades in the phylogenetic tree of *A. terreus*. The first clade was constituted by *A*.* terreus* NIH2624, 45A, ATCC 20541, M6925, w25, ASM-1, ML-44, and T3 Kankrej strains. The second was *A*. *terreus* ATCC 20542, and the third was by *A*. *terreus* IFO 6365 and TN-484 (Supplementary Fig. S4). *A. terreus* T3 Kankrej showed the highest number of contigs/scaffolds and the lowest N50 value. We evaluated the completeness of *A*. *terreus* strains using BUSCO. BUSCO analysis indicated that the genome assembly contained > 95% complete single-copy BUSCOs and < 0.03% missing BUSCOs in all *A*. *terreus* strains, except for *A*. *terreus* T3 Kankrej with < 40% complete single-copy BUSCOs and > 40% missing BUSCOs (Supplementary Fig. S5). Therefore, we excluded *A*. *terreus* T3 Kankrej from subsequent analyses as the quality of genome assembly was unlikely sufficient for later analyses^27^.

### The CAZymes and CYPs analysis among *A. terreus* strains

**Figure 1:**
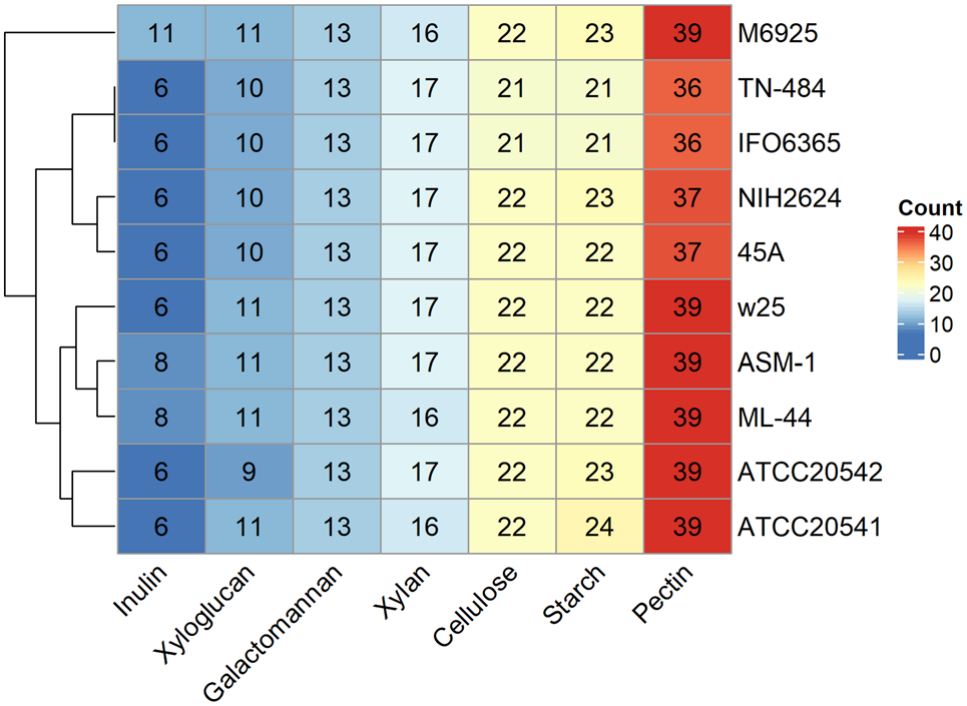
Number of genes related to degradation of different plant-based polysaccharides detected in *A. terreus* genomes.

To evaluate the plant biomass-degrading ability across all *A. terreus* strains, the genes from the CAZyme family were predicted using dbCAN2 meta server with HAMMER, DIAMOND, and eCAMI^38^. CAZymes were divided into six main groups: glycoside hydrolases (GH), glycosyltransferases (GT), polysaccharide lyases (PL), carbohydrate esterases (CE), auxiliary activities (AA), and carbohydrate-binding modules (CBMs). According to De Vries et al.^39^, seven CAZymes families (cellulose, xylan, galactomannan, xyloglucan, pectin, starch, and inulin) were found in the plant biomass-degrading ability of *Aspergillus* spp. (Supplementary Table S2). All strains showed the highest and lowest percentage of value of genes associated with pectin degradation and inulin degradation, respectively. *A. terreus* M6925 showed 135 plant biomass-degrading related genes, and it was different from the other strains. The number of CAZymes genes in *A. terreus* ATCC 20541 was similar to those in *A. terreus* ATCC 20542 (Fig. 1). Fungal CYPs are associated with diverse biosynthesis including the production of primary and secondary metabolites and denitrification of xenobiotics^40^. In the CYP results, *A. terreus* ATCC 20541 had 150 CYPs, which could be classified into 22 families. The CYP52 family contained the highest number of genes (40–44 genes), and the second largest group including CYP504 and CYP58 family contained 15–22 genes in *A. terreus* strains (Table 2).

**Table 2:** Comparison of the cytochrome P450 genes in the *A. terreus* strains.

CYP	ATCC 20541	ATCC 20542	45A	ASM-1	ML-44	NIH2624	w25	M6925	IFO 6365	TN-484
CYP52	41	40	40	40	40	41	41	42	44	44
CYP504	18	18	18	18	18	18	18	22	18	18
CYP58	15	19	14	17	17	19	19	21	18	18
CYP3	11	12	12	13	12	11	11	12	11	11
CYP51	11	11	13	10	10	13	10	10	7	7
CYP65	10	10	12	11	11	10	10	14	13	13
CYP61	9	9	6	9	9	9	6	9	6	6
CYP68	9	6	4	7	7	6	6	6	6	6
CYP5293	4	5	5	7	7	4	7	7	3	3
CYP620	3	3	3	3	3	3	3	3	3	3
CYP5080	3	2	3	2	2	2	2	2	2	2
CYP505	3	2	2	2	2	2	2	2	2	2
CYP102	3	2	2	3	3	3	4	3	3	3
CYP82	2	2	2	2	2	2	2	2	3	2
CYP83	1	1	1	1	1	1	1	1	1	1
CYP541	1	1	1	1	1	1	1	1	1	1
CYP53	1	1	1	1	1	1	1	1	1	1
CYP2	1	1	1	1	1	1	1	4	2	2
CYP78	1	1	1	1	1	1	1	1	1	1
CYP71	1	1	1	1	1	1	1	1	1	1
CYP98	1	1	1	1	1	1	1	1	1	1
CYP4	1	1	1	1	1	1	1	1	1	1

### Secondary metabolism and amino acid variants

**Figure 2:**
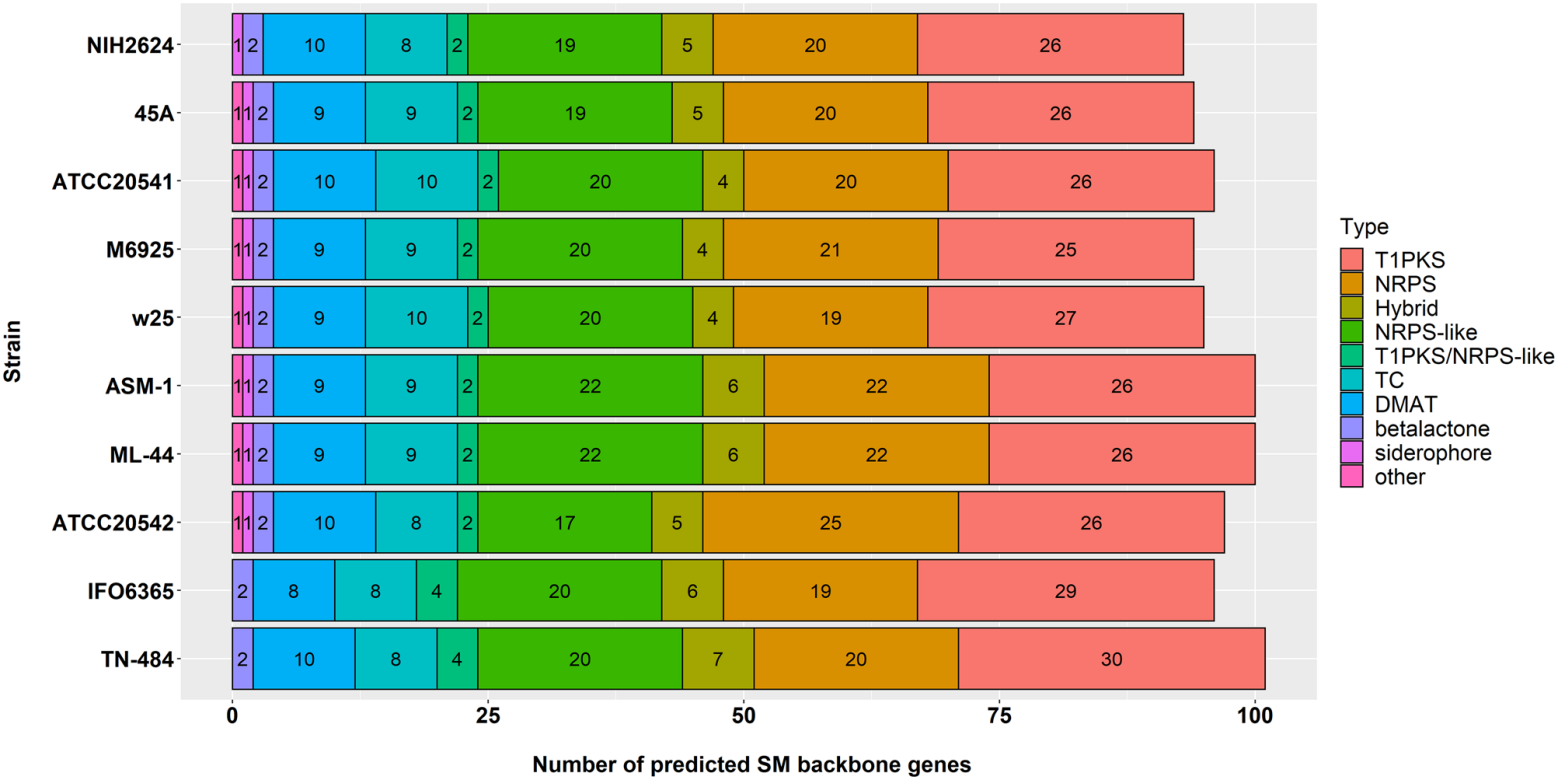
Core biosynthetic genes of *A. terreus* strains predicted by the antiSMASH database based on AUGUSTUS annotations. *T1PKS* polyketide synthase, *NRPS* nonribosomal peptides synthase, *Hybrid* PKS-NRPS hybrid, *TC* terpene cyclase, *DMAT* dimethylallyl tryptophan synthase.

*A. terreus* ATCC 20541 genome encoded 73 SM biosynthetic backbone genes according to the fungal antiSMASH v 6.0.1 database^41^. SM biosynthetic backbone genes types in *A. terreus* strains (Fig. 2) included type I polyketide synthase (T1PKS), non-ribosomal peptide synthetase (NRPS), PKS-NRPS hybrid, NRPS-like, T1PKS/NRPS-like, terpene (TC), indole (DMAT), betalactone, and siderophore. T1PKS were richly represented among *A. terreus* strains; and the itaconic acid producers, such as IFO 6365 and TN-484, showed the highest number of putative T1PKS. Additionally, *A. terreus* IFO 6365 and TN-484 lacked the SM cluster type of siderophore (Fig. 2).

**Figure 3:**
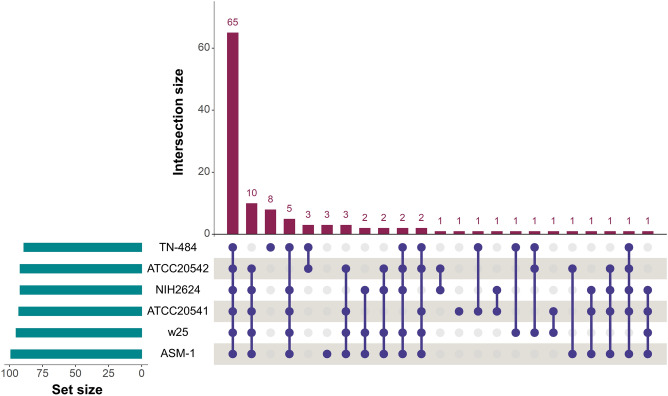
Comparison of the predicted secondary metabolism in *A. terreus* strains. Upset plot showing SM proteins from six representative genomes in each clade.

**Figure 4:**
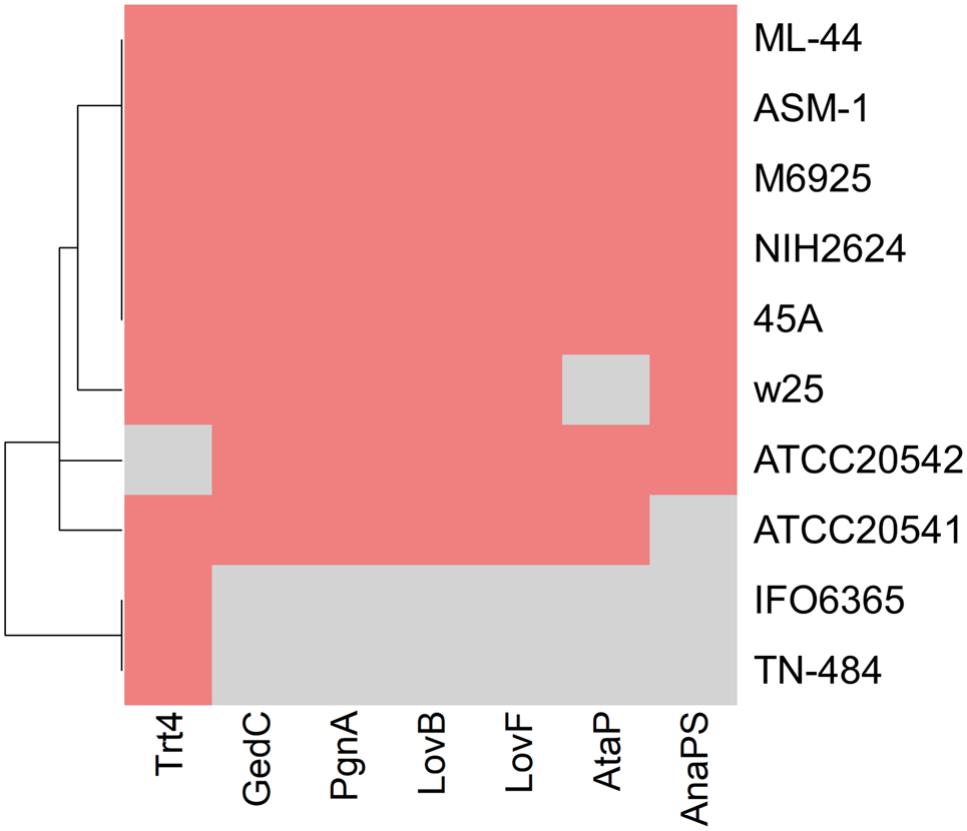
Heatmap represents the presence (pink) or absence (gray) matrix of orthologous *A. terreus* NIH2624 core biosynthetic proteins associated with SMs in *A. terreus* strains. The dendrogram was generated based on hierarchical clustering analysis. X-axis: orthologous *A. terreus* NIH2624 core SM proteins (Romsdahl and Wang, 2019); Y-axis: strain clustering. The completed results were given in Supplementary Fig. S7.

We observed 65 conserved core SM proteins among six *A. terreus* strains, and 20 SM proteins among five *A. terreus* strains. *A. terreus* ATCC 20541 was predicted to encode a unique SM protein, which was similar to M6925 (Fig. 3; Supplementary Fig. S6). *A. terreus* TN-484 and ASM-1 were predicted to encode eight and three unique SM proteins, respectively. We noted that *A. terreus* TN-484 has the highest number of unique SM proteins among all strains, and *A. terreus* IFO 6365 and TN-484 were the most phylogenetically distant from other *A. terreus* strains (Fig. 3; Supplementary Fig. S6). Interestingly, our analyses indicated that the genome of *A. terreus* ATCC 20541, ATCC 20542, and w25 did not encode non-ribosomal peptide synthetase (AnaPS), terretonin (Trt4), and acetylaranotin (AtaP), respectively (Fig. 4; Supplementary Fig. S7).

### Lovastatin BGC comparison with *A. terreus*,* M. pilosus* and *P. citrinum*

**Figure 5:**
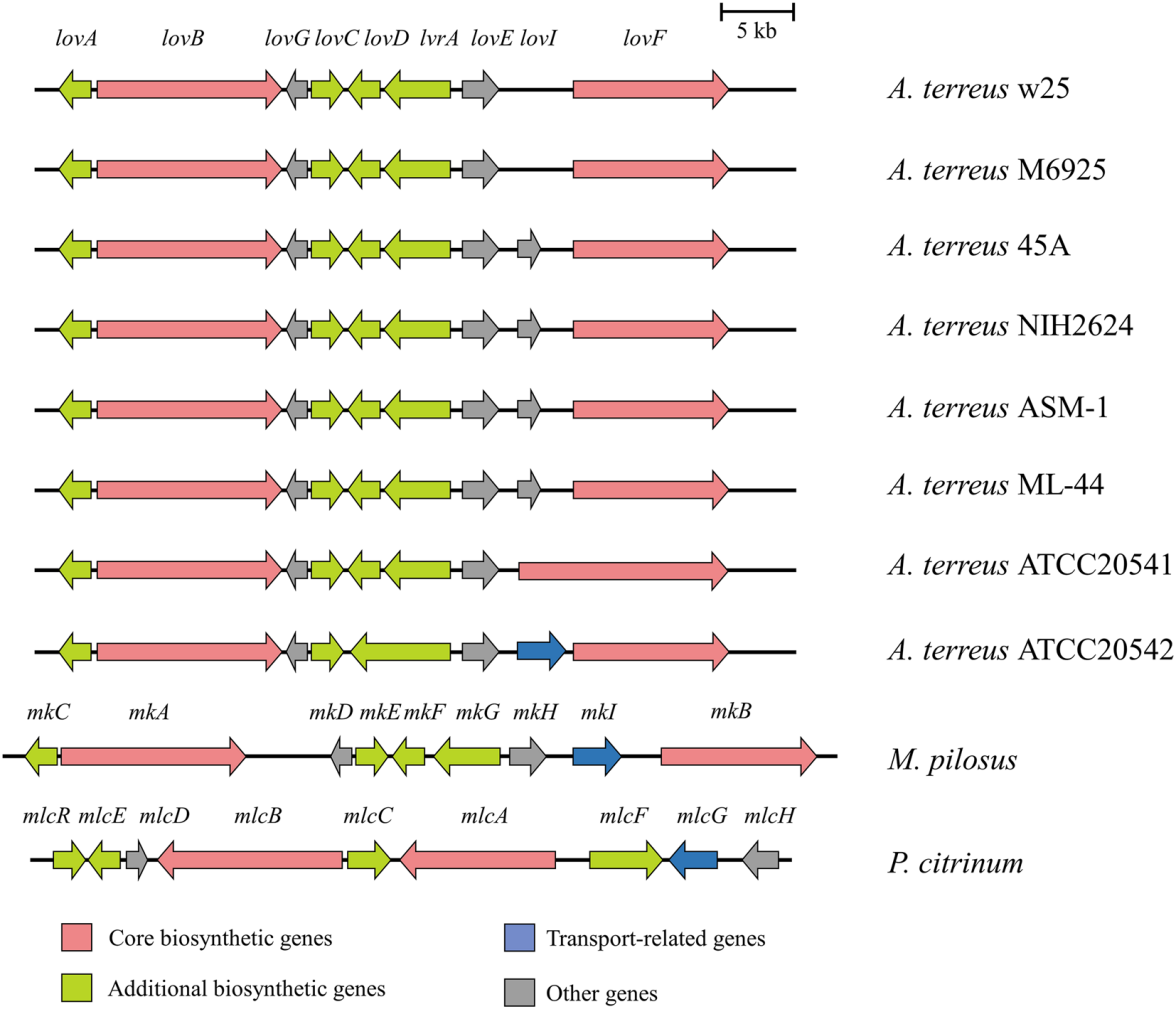
Genes involved in the biosynthesis of lovastatin (*A. terreus* strains), monacolin K (*M. pilosus*), and compactin (*P. citrinum*).

**Figure 6:**
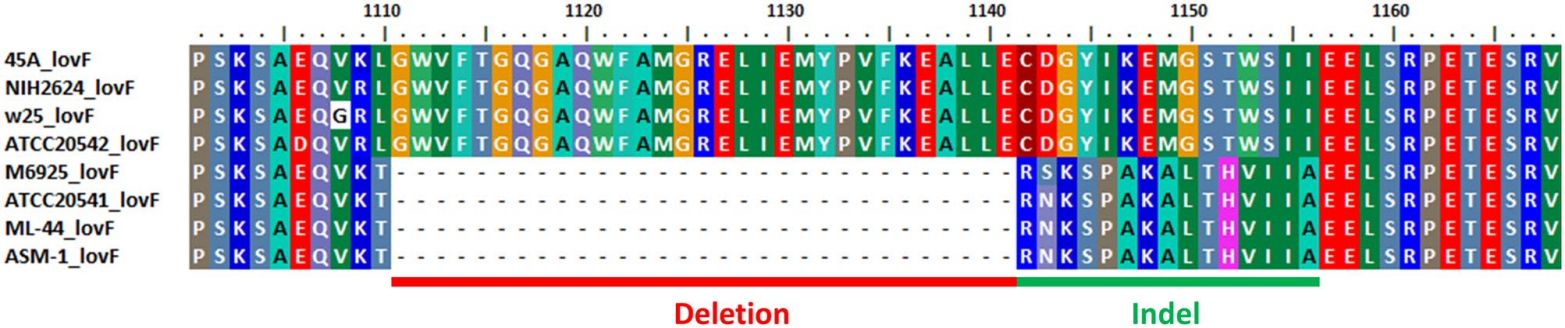
Sequence alignment showing the amino acid sequence coded by *lovF*. A deletion and an indel region were observed. *A. terreus* M6925, ATCC 20541, ML-44, and ASM-1 lacked 31 amino acids (red line) and had 15 indels (green line) in *lovF* (position: 1111–1156).

Several filamentous fungi produce lovastatin, such as *Monascus* species (*M*. *ruber*, *M. purpureus*, *M*. *sanguineus*, and *M*. *pilosus*), *Penicillium citrinum* (*P*. *citrinum*), *Trichoderma viride* etc^42–45^. The lovastatin BGC comparison showed that *lovI* was absent in *A. terreus* w25 and M6925 strains, and *lovI* was linked with *lovF* in *A. terreus* ATCC 20541. In *A. terreus* ATCC 20542 *lovI* acts as a transport-related gene, but in *A. terreus* NIH2624, ASM-1, ML-44 and 45A *lovI* act as the “other genes” (note: the term “other genes” was shown in antiSMASH v. 6.0.1 database). In addition, the arrangement and composition of lovastatin BGC in *A. terreus* ATCC 20541, ASM-1, ML-44, ATCC 20542, NIH2624, 45A, M6925, and w25 were similar to the BGC in *M. pilosus* and *P. citrinum* (Fig. 5). *LovB* and *lovF*, the “core biosynthetic genes”, play crucial roles to form the lovastatin core structure^46,47^. 30 and 59 amino acid changes (referred to as a non-synonymous single nucleotide polymorphism (SNP) or mutation) were observed in *lovB* and *lovF* genomic regions in *A. terreus* ATCC 20541 (Supplementary Figs. S8, S9). There were 115 transpositions, including 84 additional amino acids in positions 395–566 and 31 missing amino acids in positions 1111–1141, and 15 indels in positions 1142–1156, were observed in *lovF* of *A. terreus* ATCC 20541, ML-44, and ASM-1 (Fig. 6; Supplementary Fig. S9).

## Discussion

In this study, we present a high-quality whole genome assembly of *A. terreus* ATCC 20541 containing 10,410 protein coding genes with a total genome size of ~ 30 Mb. The largest scaffold size was 3.82 Mb and the N50 value of scaffolds was 1.58 Mb. In addition, all publicly available whole genome sequences of *A. terreus* strains were retrieved and further compared. The total genome size (28.0–31.8 Mb) and GC content (52.1–53.1%) of eleven *A. terreus* strains are similar to all *Aspergillus* genomes (genome sizes are 29–41 Mb and GC contents vary from 43 to 53%)^36,39^. Phylogenetic analysis indicates that *A. terreus* ATCC 20541 is positioned in a major clade including *A. terreus* NIH2624, 45A, M6925, w25, ASM-1, ML-44, and T3 Kankrej, so the genomic relationship in *A. terreus* ATCC 20541 is closely related to most *A. terreus* strains, especially *A. terreus* NIH2624 and 45A. Most *A. terreus* strains are divergent from *A. terreus* ATCC 20542, IFO 6365, and TN-484 (Supplementary Fig. S4).

Several *Aspergillus* spp. can degrade plant biomass polysaccharides which is a major carbon source for many fungal species. The fungal CAZymes family has been utilized in industrial applications by the hydrolysis of plant biomass for the subsequent production of biofuels and high-value biochemicals^48^. Our results indicate that *A. terreus* strains secrete high levels of pectinases, so they are capable of degrading pectin which is a complex polysaccharide in plant cell walls^49^. Abundant genes in GH31 (starch), GH32 (inulin), GH28 (pectin), GH78 (pectin), PL1 (pectin), and AA9 (cellulose) of the CAZymes family were observed, which ranged from 10 to 11, 6 to 11, 7 to 8, 5 to 7, 7, and 10 genes, respectively (Supplementary Table S2).

Fungi have diversified CYP450 families, which contribute to survival strategies, to adapt to varying environments, and to facilitate detoxification^50^. The CYP52 family is the largest (40 to 44 genes) in the *A. terreus* strains (Table 2). This CYP takes part in degrading n-hexadecane (HXD) when it is used as the sole carbon source in *Aspergillus* sp. RFC-1^51^. Previous studies reported that the CYP504 family including 239 proteins can catalyze phenylacetate catabolism and may be related to the diversified functions of xenobiotic compounds detoxification^52,53^. In addition, the CYP58 family including 274 proteins in *A. terreus* strains was related to aflatoxin biosynthesis, and the CYP58 family also can be found in *A. flavus* and *A. parasiticus*v^54^. CYP51 family (CYP51A, CYP51B, and CYP51C) including 720 proteins in *A. flavus* is an antifungal drug target for controlling pest and fungal plant diseases^50^. Interestingly, the CYP51 family, the target of azole drugs, was found in different *A*. *terreus* strains in this study. Several studies have already detailed the role of CYP51 variation in azole resistance in *A*. *terreus*^55^ and have identified three CYP51 paralogs^56^.

Apart from *A. terreus* IFO 6365 and TN-484, other *A. terreus* strains contained the BGC of siderophore production. Siderophore is one of the metabolites secreted by *A. terreus*^57^ and *A. fumigatus*^58^. These metabolites sequester iron from the host microbes and cycle this metal nutrient to themself^59^. In addition, *A. terreus* IFO 6365 and TN-484 are itaconic acid producers, and LovF, LovB, GedC, PgnA, AtaP, and AnaPS are not found in their core of SM proteins. *A. terreus* ATCC 20541 lacks an SM AnaPS, which participates in acetylaszonalenin production^60^. *A. terreus* strains produce several interesting bioactive compounds such as butyrolactones (BtyA), asterriquinone (AtqA), terreic acid (AtX), citreoviridin (CtvA), terrein (TerA), Trt4, and geodin (GedC) (Supplementary Fig. S7), which could be potential sources of biosynthetic enzymes, antibiotics, or antitumor in the future^61–67^.

We also compared the lovastatin BGC in *A. terreus* strains. The lovastatin BGC including *lovA*, *lovB*, *lovC*, *lovD*, *lovE*, *lovF*, *lovG*, *lovI*, and *IvrA* in *A. terreus* strains are similar to the monacolin K BGC in *M. pilosus* BCRC 38072 and to the compactin BGC in *P. citrinum* (Fig. 5). The hypolipidemic agents monacolin K and compactin (mevastatin) serve the same function as lovastatin, which act as competitive inhibitors of HMG-CoA reductases^68,69^. Additionally, we found that there were significantly different types of *lovI* gene variants among the *A. terreus* strains. The *lovI* gene is a hypothetical protein HFD88_005927 composed of 517 amino acid residues in *A. terreus* ATCC 20542. *lovI* gene had approximately 400 amino acid deletions in *A. terreus* NIH2624, ASM-1, ML-44 and 45A against the corresponding gene of *A. terreus* ATCC 20542. Notably, *lovD* and *lvrA* are fused into a single gene in *A. terreus* ATCC 20542, which is different from other *A. terreus* stains. A similar phenomenon also showed in *Bacillus subtilis* strains. For example, *ppsB* 3′ and *ppsB* 5′ are fused into a single *ppsB* in plipastatin BGC of *B. subtilis*, which could inhibit the *Fusarium* species (*F. oxysporum* and *F. graminearum*)^70^. This mechanism in *A. terreus* still requires further investigations.

In conclusion, a high-quality whole genome assembly of *A. terreus* ATCC 20541 was presented in this study. Compared with genomic sequences among *A. terreus* strains, diversified conserved BGCs were identified. Amino acid deletions and indels observed in the *lovB* and *lovF* genes played an important role in the lovastatin formation. Our comparative analyses may motivate further investigation to study the function of BGCs associated with valuable SMs, and understand the genomic diversity in *A. terreus*.

## Methods

### Strains and genomic DNA isolation

*A. terreus* ATCC 20541 used in this study was collected from American Type Culture Collection (Manassas, VA, USA). The strain was cultured on potato dextrose agar (PDA) at 28 °C. The *A*. *terreus* ATCC 20541 genomic DNA was extracted as described in Aamir^71^ with the following modifications. First, the harvested fungal tissue was homogenized in lysis buffer (50 mM EDTA, 3% SDS, 100 mM Tris–HCl, and pH 8). The homogenate was centrifuged at 13,000 rpm for 10 min, and the supernatant was then mixed with an equal volume of phenol: chloroform: Isoamyl alcohol (25:24:1). After centrifugation, the aqueous layer was collected in a new eppendorf tube, followed by ethanol precipitation and centrifugation. The DNA pellet was dissolved in Tris–HCl buffer, pH 8.5. The genomic DNA quality was assessed on an Agilent 2100 Bioanalyzer (Agilent Technologies, USA), and DNA integrity was checked using 1% agarose gel electrophoresis then for sequencing by Illumina sequencing platform.

### Genome sequencing, assembly and quality assessment

The DNA library was constructed using the Illumina TruSeq Nano DNA High Throughput Library Prep Kit (Illumina, USA) according to the manufacturer’s instructions. Whole-genome shotgun sequencing was performed using Novaseq 6000 platforms (Illumina, USA) with 2 × 150 paired-end reads. The raw reads were trimmed by removing adaptor sequences and low-quality sequences with Q < 20 using Trimmomatic v.0.39 with parameters of “SLIDINGWINDOW:4:20”^72^, and MultiQC v.1.2^73^ summarized the sequence of quality control. The de novo genome assembly was carried out with SPAdes v.3.10.1^74^, and the assembly was polished with Pilon v1.23^75^. The assembly was evaluated using QUAST v.4.5^76^, and the gVolante was used to assess the completeness of the genome assembly, according to the Eurotiomycetes database^77^.

### Genome annotation

Genome assemblies of ten *A. terreus* strains were obtained from NCBI (access date: November 2021). All the *A. terreus* strains were annotated using AUGUSTUS v. 3.4.0^78^ with “*Aspergillus terreus*” as a training dataset, and the parameters were based on Takahashi et al.^29^. Ribosomal RNA (rRNA) and transfer RNA (tRNA) genes were predicted using barrnap v. 0.9 and tRNAscan-SE v. 2.0.9^79^. Signal peptide and transmembrane helices were functionally annotated using SignalP v. 5.0b^80^ and Phobius server^81^ (access date: November 2021). The predicted proteins were assigned by the eukaryotic orthologous group (KOG) using the webMGA server^82^ with a cut-off e-value ≤ 1e − 5. KEGG Automated Annotation Server (KAAS) was used for pathway mapping of Eurotiomycetes species with a bi-directional best hit (BBH)^83,84^. Gene ontology (GO) was predicted using PANNZER web server^85^. Proteinortho program v. 6.0.31^86^ with “blastp” function was used to detect orthologous genes within the *A. terreus* strains.

### CAZymes and CYP family in *A. terreus* strains

CAZymes annotation was performed using dbCAN2 meta server^38^ (access date: November 2021) with HAMMER, DIAMOND, and eCAMI. The cytochrome P450 gene family classifications in *A. terreus* strains were performed based on *A. terreus* NIH2624 using biocatnet CYPED v 6.0^87^ with cut off e-value ≤ 1e − 5 and identity > 40%.

### Construction of phylogenetic trees

Sequence similarity, the Average Nucleotide Identity (ANI) values of *A. terreus* strains were calculated using PYANI v.0.2.11 based on BLAST + program^88^. The phylogenetic tree of *A. terreus* strains was constructed using R language.

### Secondary metabolite gene clusters and amino acid variants detection

The whole genome sequence of *A. terreus* strains was analyzed by using the antiSMASH v. 6.0.1 (fungal version)^41^ with default parameters to identify the potential secondary metabolite gene clusters. Amino acid variants were identified by pair-wise alignment of conservation gene using ClustalW in Bioedit v. 7.2.6.

### Ethics approval and consent to participate

This study does not contain any experiments with human and animals performed by all the authors.

## Supplementary Information

Supplementary Figures.

Supplementary Table S1.

Supplementary Table S2.

## Data Availability

The Whole Genome Shotgun project has been deposited at GenBank in NCBI. The accession number is JANHGT000000000 and BioProject is PRJNA861866. The deposited data will be released upon acceptance. The submission confirmation letter sent by GenBank NCBI is attached in the related file.
