# Supplementary material for: Whole genome sequence characterization of Aspergillus terreus ATCC 20541 and genome comparison of the fungi A. terreus
Source: Sci Rep. 2023 Jan 5;13:194. doi: 10.1038/s41598-022-27311-7 (PMC9814666; doi:10.1038/s41598-022-27311-7)
Supplement: Supplementary file 1 — Supplementary Figures. [file 41598_2022_27311_MOESM1_ESM.pdf]

Supplementary

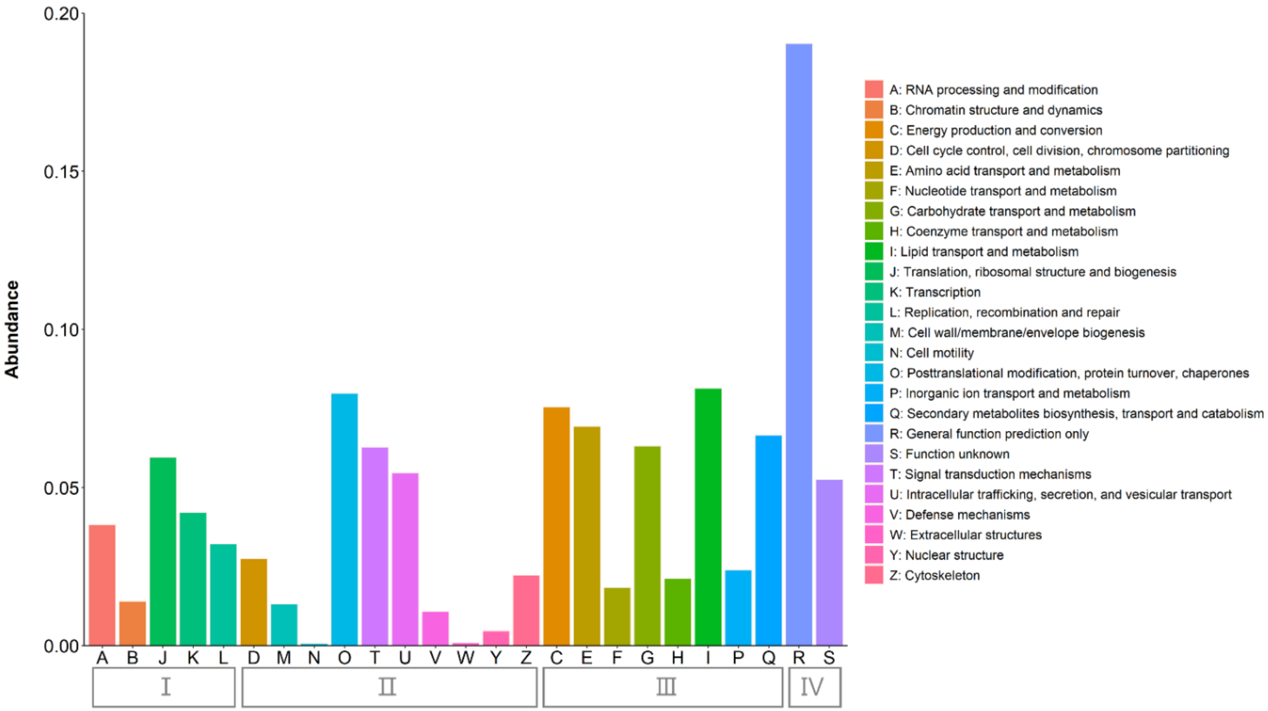

Figure S1. Histogram of KOG distribution of predicted proteins from *A. terreus* ATCC 20541.

A total of 3,191 KOG groups were assigned to 6,689 *A. terreus* proteins using the webMGA tool with a cut-off e-value of 1e −5. KOG function classification was divided into 26 classes (A-Z), which were assigned to four major groups including classification information storage and processing (I), cellular processes and signaling (II), metabolism (III), and function poorly characterized proteins (IV).

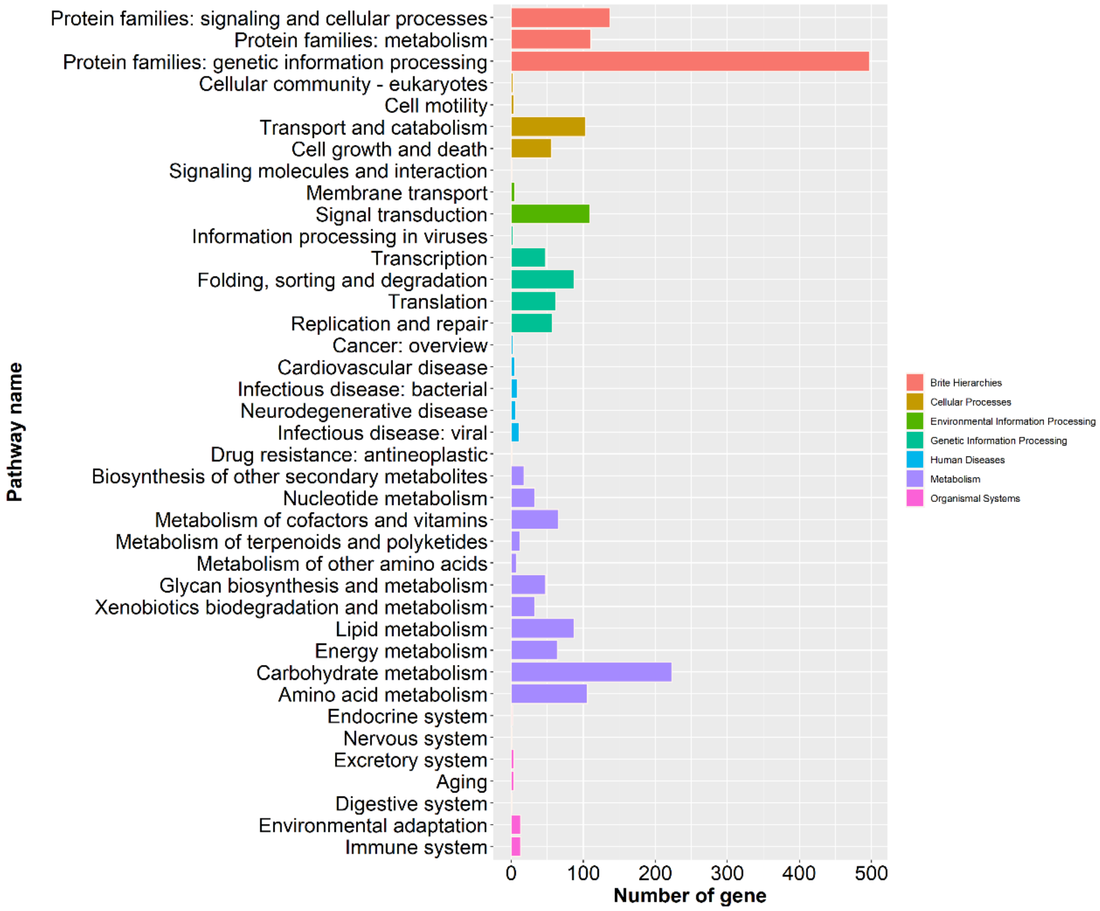

Figure S2. KEGG pathway analysis of *A. terreus* ATCC 20541 genome.

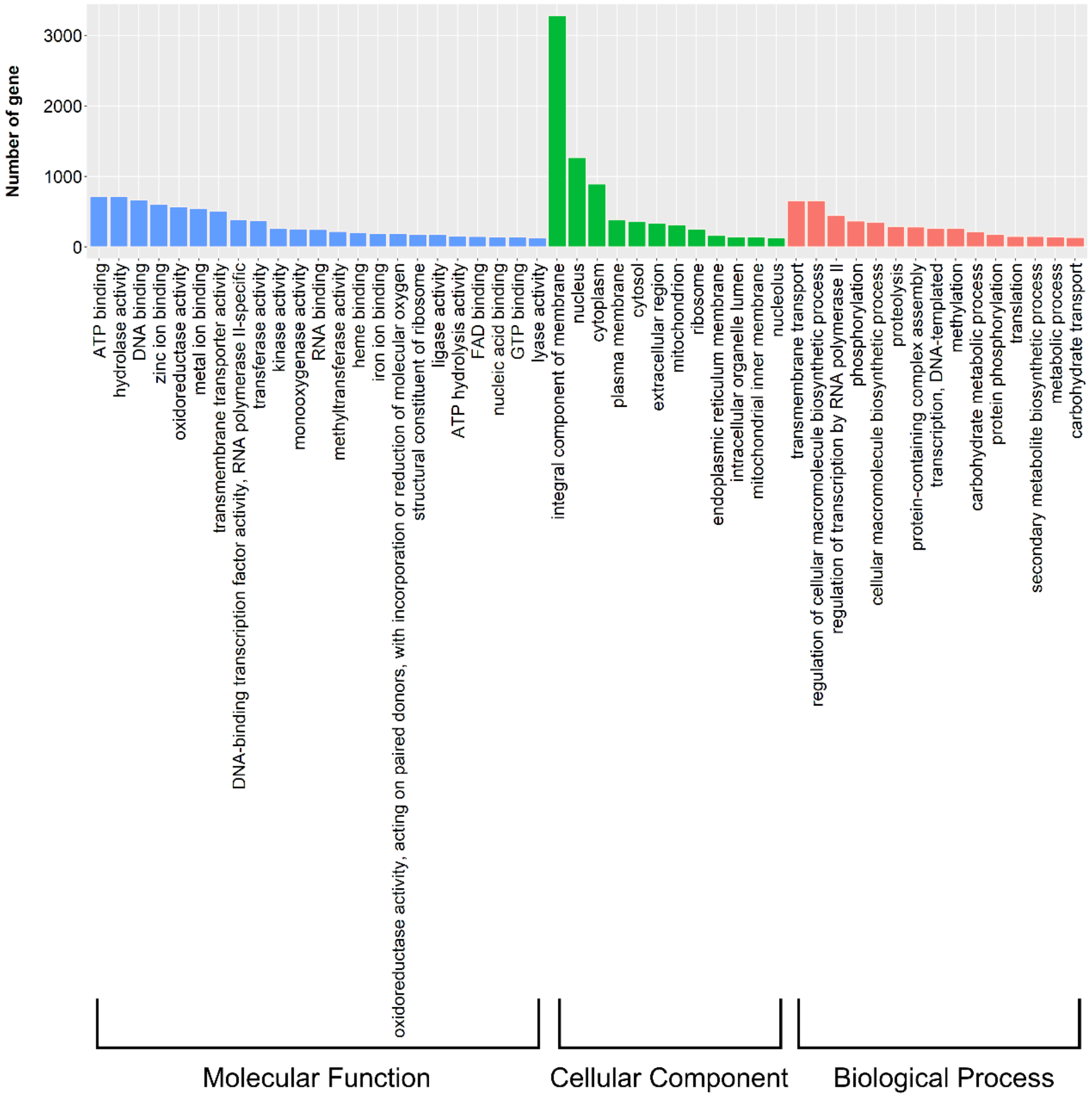

Figure S3. GO analysis of *A. terreus* ATCC 20541 genome (top 50).

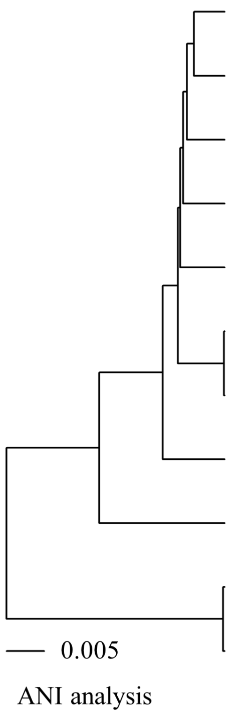

Strain	Total genome size (Mb)	Number of contigs/ scaffolds	N50 (bp)	GC (%)	Isolation source	Accession no.	Reference
NIH2624	29.4	27	1,912,493	52.9	A patient isolate	GCA_000149615.1	-
45A	29.3	26	1,912,382	53.0	Soil, India	GCA_001630395.1	Savitha et al., 2016
ATCC 20541	30.0	156	1,581,806	52.3	ATCC, USA	-	This study
M6925	31.8	35	4,133,337	52.2	Saint James Hospital Dubline, Ireland	GCA_009834425.1	Palanivel et al., 2020
w25	29.4	536	326,294	52.7	Inner root, Brazil	GCA_002749855.1	-
ASM-1	30.7	199	1,140,755	52.4	Mutant of ML-44	GCA_015266375.1	Wu et al., 2021
ML-44	30.9	111	1,671,991	52.3	The gut of pacific oyster, China	GCA_015333565.1	Wu et al., 2019
T3 Kankrej	28.0	13,340	3,497	52.1	Rumen, India	GCA_002930435.1	-
ATCC 20542	30.4	9	4,253,827	52.2	ATCC, USA	GCA_016808415.1	Ryngajłło et al., 2021
TN-484	29.7	24	1,952,528	52.5	Mutant of IFO6365	GCA_009014675.2	Kanamasa et al., 2019
IFO 6365	28.7	23	2,163,102	53.1	-	GCA_009932835.1	Takahashi et al., 2020

Figure S4. Characteristics of complete *A. terreus* genomes deposited at the NCBI database.

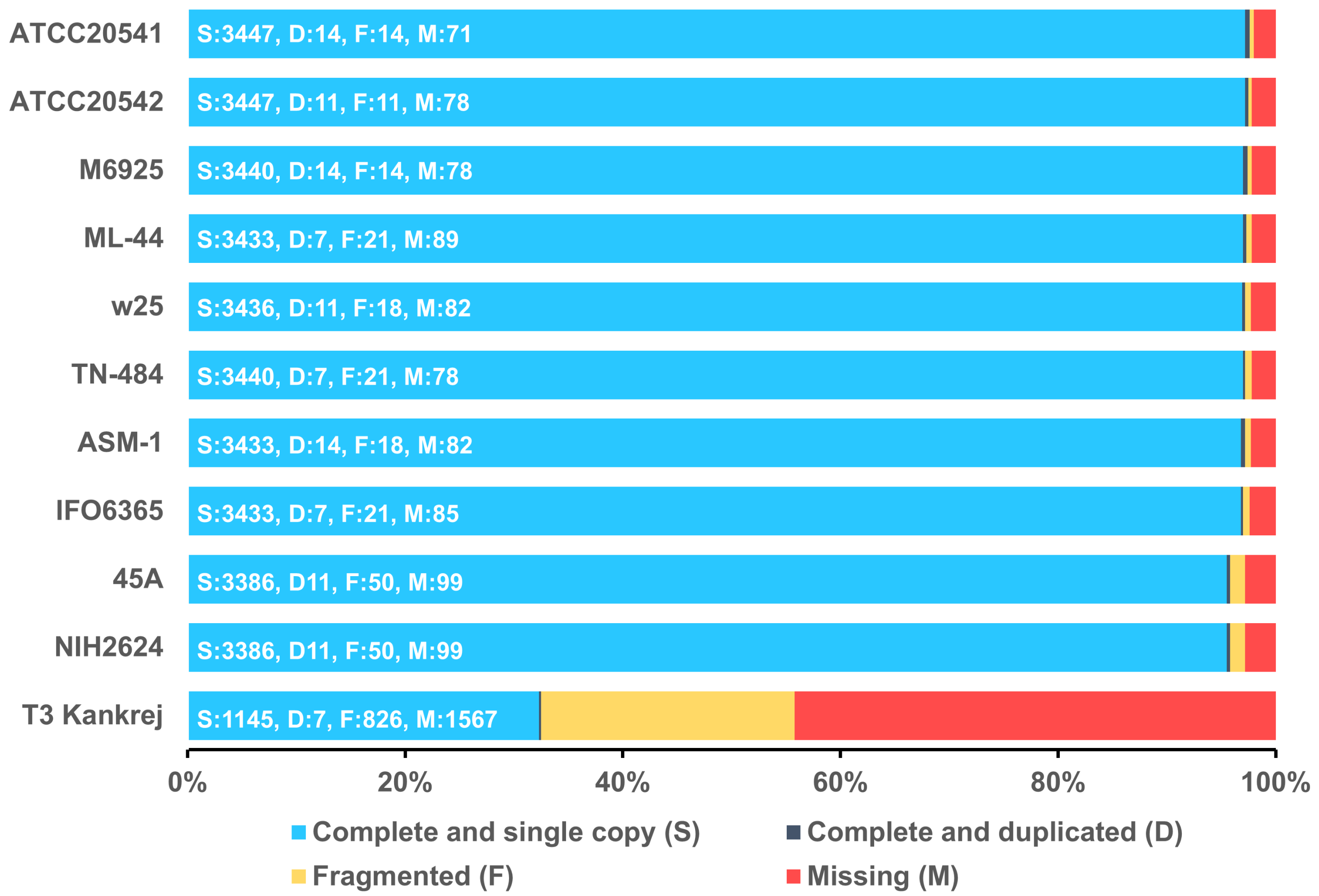

Figure S5. Genome BUSCO assessments for eleven *A. terreus* strains.

S: Complete and single-copy BUSCOs; D: Complete and duplicated BUSCOs; F: Fragmented BUSCOs; M: Missing BUSCOs.

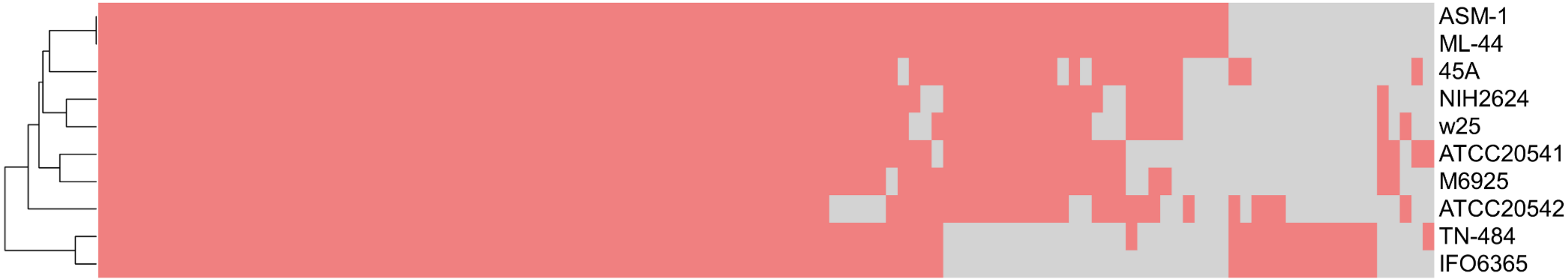

Figure S6. Comparison of the orthologous secondary metabolism in *A. terreus* strains.

Heatmap represents the presence (pink) or absence (gray) matrix of orthologous SM proteins predicted by antiSMASH v. 6.0.1 (fungal version) (Blin et al., 2021). The dendrogram was generated based on hierarchical clustering analysis. X-axis: orthologous SM proteins; Y-axis: strain clustering.

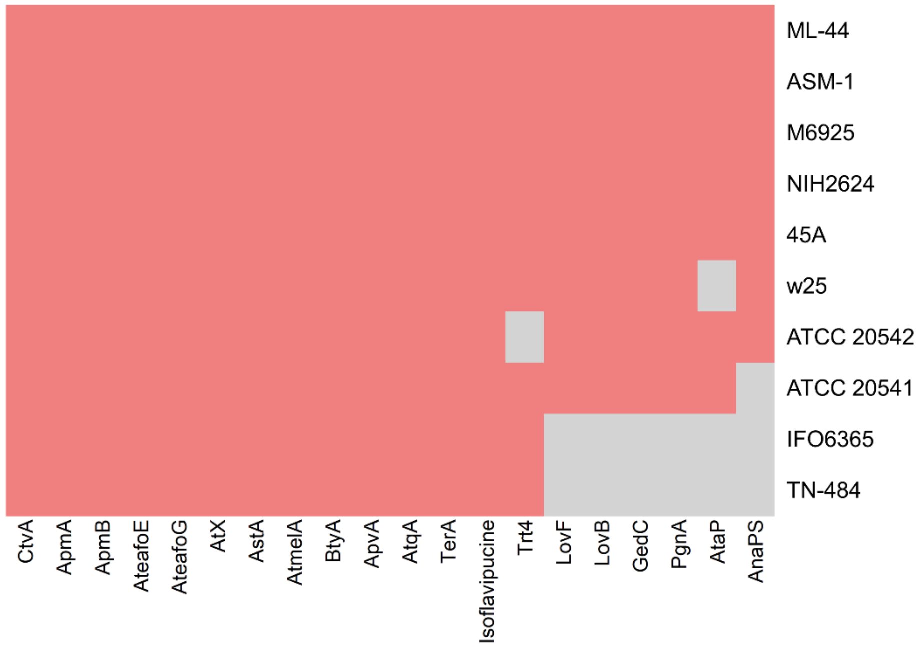

Figure S7. Comparison of the core SM proteins in *A. terreus* strains.
Heatmap represents the presence (pink) or absence (gray) matrix of orthologous *A. terreus* NIH2624 core biosynthetic proteins associated with SMs in *A. terreus* strains. X-axis: orthologous *A. terreus* NIH2624 core SM proteins (Romsdahl and Wang, 2019); Y-axis: strains.

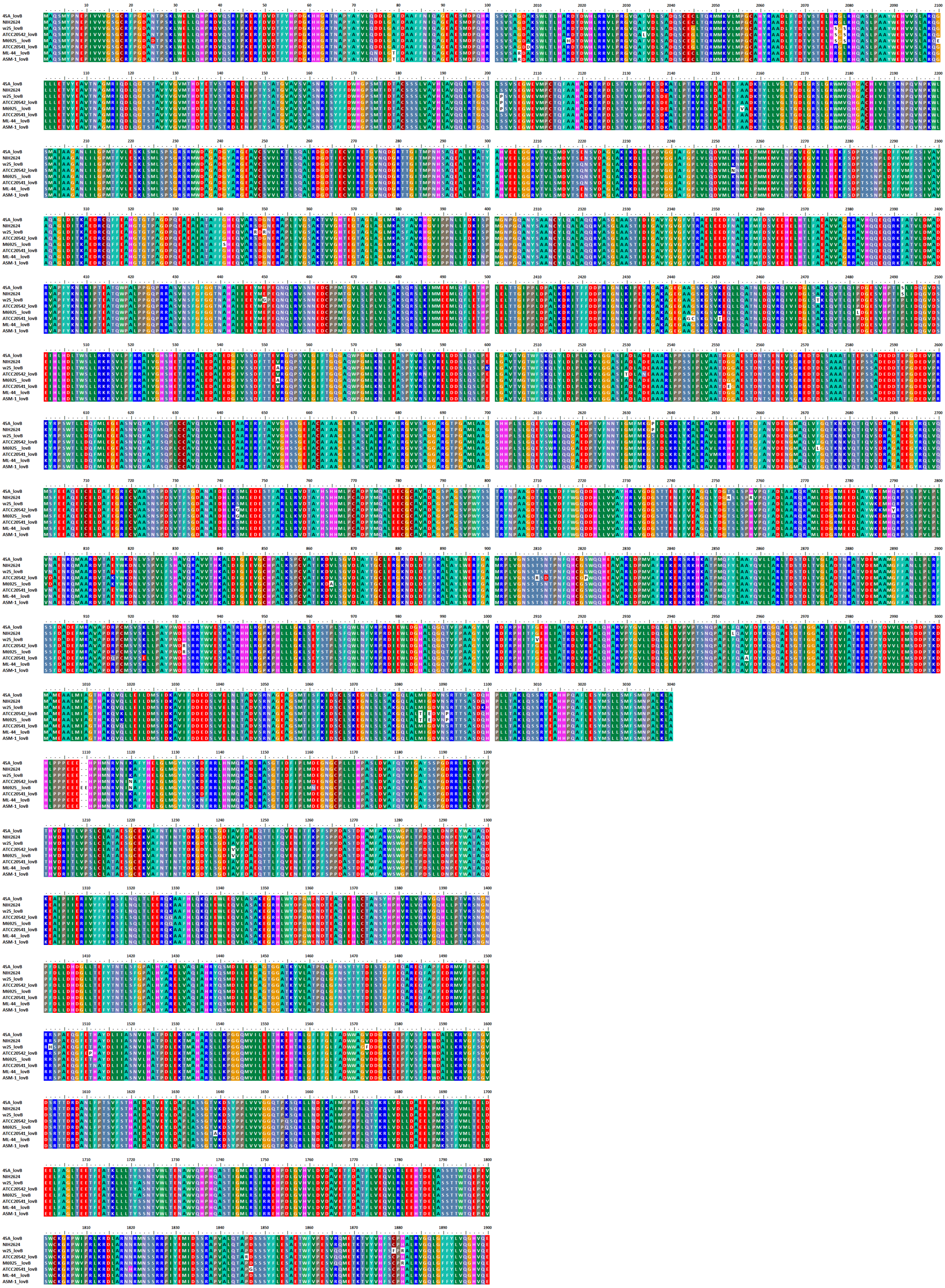

Figure S8. Sequence alignment of the *lovB* gene in *A. terreus* strains.

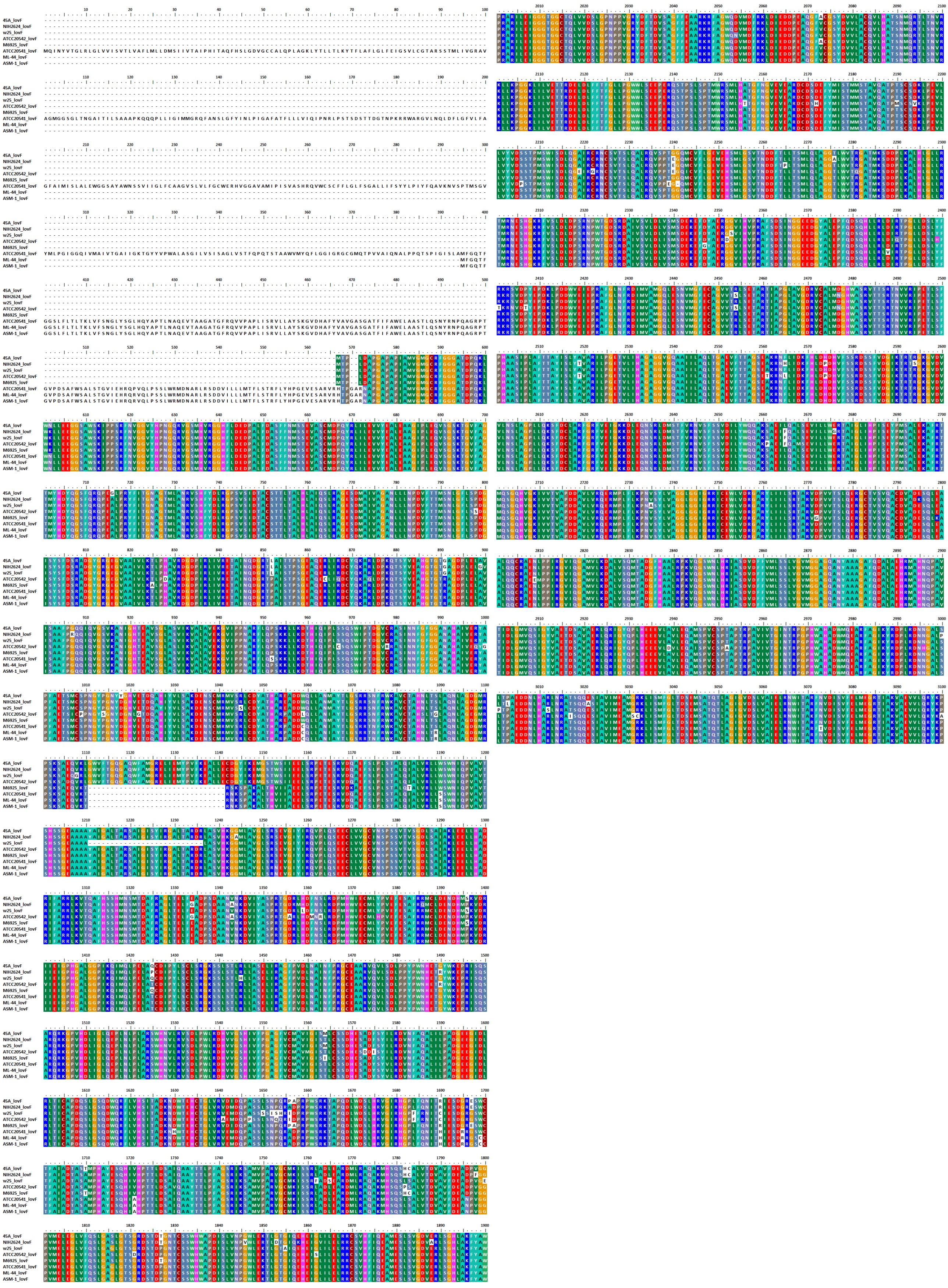

Figure S9. Sequence alignment of the *lovF* gene in *A. terreus* strains.
